# Medical students' self-report of mental health conditions

**DOI:** 10.5116/ijme.4ed1.d1e0

**Published:** 2012-01-02

**Authors:** Rael D. Strous, Netta Shoenfeld, Avi Lehman, Aharon Wolf, Leah Snyder, Ori Barzilai

**Affiliations:** 1 Beer Yaakov Mental Health Center, Sackler Faculty of Medicine, Tel Aviv University, Israel; 2 Sackler Faculty of Medicine, Tel Aviv University, Israel

**Keywords:** Medical students, subsyndromal mental illness, mental health conditions, self-evaluation, Israel

## Abstract

**Objectives:**

To investigate the subjective presenceof a range of subsyndromal and syndromal mental health conditions in medical students, and to compare the presence of these conditions between preclinical and clinical training.

**Methods:**

A cross sectional study was used among first-and fifth-year medical students. Student reported their mental health conditions using the Diagnostic and Statistical Manual of Mental Disorders criteria, the fourth version (DSM-IV). Data analysis was based on 110 questionnaires.

**Results:**

A total of 61 students (55.5%) reported that they experienced symptoms of mental illness, albeit many with minimum severity. More than 50% of the students reported that they experienced Axis I and Axis II disorders, which mostly were mood disorders (38% in year 1 and 35% in year 5) and obsessive-compulsive traits (41% in year 1 and 46% in year 5), respectively. The least common disorders reported were psychotic disorders (5% in year 1 and 0% in year 5) and schizotypal traits (7% in year 1 and 2% in year 5). Fifth-year students reported more Axis I disorders than first-year students. Female students reported more Axis I disorders than their male peers. A further analysis indicated that there was no significant association between age and Axis disorders. Several conditions were comorbid with other mental illnesses.

**Conclusions:**

A great number of students reported that they experience mental health conditions with minimal severity. This implies a need for indispensable ongoing support programs for the special needs of medical students.

## Introduction

Increasing attention is being paid to mental health care concerns of medical students –a complex issue which may be associated with training-related stressors.^1^ Several reports have described increased prevalence of mental illness in medical students. For example several studies have indicated increased rates of anxiety and depression, especially in females,^2^^,^^3^ compared to the general population.^3^ Baseline depression upon entering medical school was reported to be consistent with the general population, but an increase in depression was documented at years 2 and 4,^3^ with depression incidence during the first two medical school years estimated to be 3-fold higher than the general population.^4^ One study reported increased rates of anxiety and depression in both medical school and upon graduating from medical school^5^ with anxiety and depressive symptoms highest in the 3rd year of medical school.^6^ Risk factors for depression included prior depressive episodes and family history of depression.^4^ One-year prevalence of suicidal ideation during medical school has been reported to be 14.2% which is higher than the 2.3% prevalence in the general population, with no gender difference (consistent with general population).^7^ Interestingly in this latter study the rate of suicide attempts was lower than the general population.^7^ One relatively small study analysed personality disorders in medical students but found no significant difference when compared to the general population.^8^ Others have reported high incidence of obsessive-compulsive disorder (OCD) symptoms in the first year of medical school which progressively declined in subsequent years.^6^Methods used to evaluate mental illness in medical school students vary across studies with some using surveys to evaluate perceived mental illness^2^^,^^3^^,^^5^^,^^6^^,^^9^ while others have relied on student interviews,^4^ results of the former being constrained due to difficulties in obtaining unbiased responses.^10^ The lack of consistency of methodology raises questions about cross-study reliability of reported findings. Only a limited number of studies have relied upon DSM-IV criteria when establishing diagnostic protocol^9^ with the majority of studies investigating only a limited spectrum of DSM-IV conditions such as anxiety and depression. Considering that stress in medical school may be a risk factor for depression in residency,^11^ depression being linked to substance abuse, suicide and impaired professional function,^12^ further evaluation is required to assess the full spectrum of subthreshold/clinical psychiatric conditions in medical school. This may assist in the prediction of future dysfunction and suffering as well as to uncover whether other conditions may also be associated with such impairment. Furthermore it would be important to compare the expression of reported mental illness conditions across the years of study as well as any gender differences and co-morbidity in order to better describe the phenomenon. The issue is relevant since personal health experiences of medical students, whether they are mental or physical concerns may contribute in a vital and often unacknowledged manner to their well-being and education.^13^Mental health among medical students is an important issue to educators and health care providers in the psychiatric field. This study aims to examine the subjective experience of students rather than merely formal testing by a battery of clinical and psychological testing. Its intention is to address the subjective presence of mental illness in medical students. This will be addressed by instructing medical students to self-evaluate themselves by means of a brief one-page anonymous questionnaire based on the DSM-IV criteria.

## Methods

### Participants

A cross sectional study was conducted at Tel Aviv University, Israel. All first and fifth years medical students were invited to participate in this study. A total of 110 medical students were involved in this study, consisting of 50.9% males and 48.1% females. Two students did not report their gender. Ages ranged between 18-30 years with a mean age of 27 (SD= 2.5). In terms of the year of study, 55 (50%) were in the first year and 49 (44.5%) were in fifth year and 6 (5.5%) were missing data. Medical students received no reward for taking part in the study.

### Instrument

We slightly modified a self-administered questionnaire for the evaluation of medical students’ mental health based on a questionnaire which has been already used for psychiatrists, social workers and psychologists.^10^^,^^14^ The questionnaire consisted of three parts. In part 1, we explained the purpose of the study and that study participation is entirely voluntary, anonymous and confidential. In part 2, we asked students to provide demographic information such as gender and year of medical study. In part 3, a 24-item rating scale was used to evaluate mental health conditions across Axis I (13 items) and Axis II (11 items) of the DSM-IV. A summary of DSMIV criteria was attached to the questionnaire in order to assist students in answering the questionnaire. This helped them to better report their own mental health conditions on the questionnaire, when we asked them to indicate whether they have ever noticed, at any time in their lives, a particular mental problem of the Axis I and II conditions. Items were evaluated on a five-point Likert scale ranging 0 (not at all a problem) to 5 (the problem is severe in degree). Therefore, the total score can range from 0 to 5 for each condition, with higher scores indicating a greater severity of condition. For the purpose of this study, a score of 3 or greater indicates the presence of a mental health condition.

### Procedures

The study was approved by the Beer Yaakov Mental Health Center Helsinki Committee Ethical Review Board. We asked each student to complete the questionnaire privately and anonymously and return it in a sealed envelope, protecting student anonymity. The students filled in the questionnaire during their breaks between classes either at least one week prior to the exam date or immediately after the psychiatry clerkship.

### Statistical Analysis

Descriptive statistical procedures were used for analysing data. We tested the association between variables using the Pearson and the point-biserial correlation coefficients, and chi-square tests. The association between gender, academic year and previous psychotherapy effects and the mental health conditions reported were analysed using t-tests. In addition, the correlation between age and the mental health conditions reported was analysed using the Pearson correlation coefficient. To avoid an inflation of alpha level (0.05) Bonferroni corrections were calculated where multiple tests were used. This led to a cut-off point of alpha=0.0035 and alpha=0.0045 for Axis-I disorders and for Axis-II traits, respectively. To assess communality between ratings of Axis-I and Axis-II students were ascribed into four groups: 1. Negative on both Axes; 2. Positive on Axis-I only; 3. Positive on Axis-II only; 4. Positive on both axes. The associations between these categories and gender and academic year were analysed using chi-square tests.

## Results

### Axis I Disorders

The most reported mental conditions were mood disorders, social phobia and sleep disorders and the least reported conditions were psychotic, sexual and impulsive disorders. The number of Axis I disorders (a score of 3 or greater) ranged between 1 and 13 per student, with an average of 2.93 disorders (SD=2.81).Table 1 shows the average rating of Axis I disorders by gender. Female students reported higher levels of mood, generalized anxiety disorders and social phobia. The total number of Axis-I disorders was more reported in female than male students. (Female=3.58±2.94 disorders; Male= 2.33±2.60 disorders; t_(106)_=2.36,p=0.02). However, these differences did not reach the corrected alpha level.

**Table 1:** Rating of Axis-I disorders among male and female students* (N=110)

Disorder	Male		Female		Statistical value		
	Mean	SD	Mean	SD	t	df	p
Mood	1.5	0.7	1.9	1.0	2.5	94	0.016
Obsessive compulsive disorder	1.5	0.8	1.6	0.9	0.2	95	0.86
Generalized anxiety disorder	1.3	0.6	1.8	1.1	2.8	95	0.007
Panic	1.2	0.8	1.5	0.9	1.4	95	0.17
Social phobia	1.4	0.9	1.8	1.1	2.1	95	0.04
Somatoform	1.4	1.0	1.6	0.9	1.2	94	0.23
Eating	1.3	0.8	1.5	0.8	1.4	96	0.18
Sexual	1.1	0.4	1.2	0.5	0.6	95	0.52
Adjustment	1.3	0.7	1.4	0.9	0.8	95	0.41
Psychotic	1.0	0.3	1.1	0.3	0.3	94	0.74
Impulse	1.2	0.6	1.2	0.6	0.3	95	0.75
Sleep	1.5	0.8	1.6	1.0	0.6	95	0.56
Substance abuse	1.3	0.7	1.2	0.6	1.1	95	0.25

* Differences did not meet the corrected alpha level

Table 2 shows the average rating of Axis I disorders in terms of medical school year. As we can see from this table, fifth year students reported higher levels of obsessive compulsive disorder and somatoform disorders in comparison first year students. The total number of Axis-I disorders was more reported in fifth year than first year students (First year students=2.02±2.58 disorders; Fifth year students= 3.73±2.58 disorders; t _(102)_ =3.38, p=0.001).

**Table 2:** Rating of Axis-I disorders among 1st and 5th year students (N=110)

Disorder	Male		Female		Statistical value		
	Mean	SD	Mean	SD	t	df	p
Mood	1.6	0.8	1.8	1.0	1.0	90	0.30
OCD	1.2	0.6	1.7	0.9	3.1	91	0.002
GAD	1.5	0.8	1.5	0.9	0.3	91	0.77
Panic	1.2	0.7	1.5	1.0	1.2	91	0.23
Social phobia	1.4	0.9	1.8	1.1	2.0	91	0.05
Somatoform	1.2	0.4	1.8	1.1	3.5	90	0.001
Eating	1.4	0.9	1.4	0.7	0.02	92	0.98
Sexual	1.1	0.4	1.1	0.5	0.6	91	0.53
Adjustment	1.3	0.7	1.5	0.9	1.1	91	0.27
Psychotic	1.1	0.3	1.0	0.0	1.4	90	0.17
Impulse	1.1	0.3	1.3	0.7	1.8	91	0.08
Sleep	1.6	0.9	1.5	0.9	0.6	91	0.53
Substance abuse	1.1	0.5	1.3	0.7	1.4	91	0.17

Nine students reported that they are under treatment for their self-reported conditions. Compared to students who did not report under treatment with a condition, these students reported higher ratings of panic disorder only (Yes=2.00±1.22; No=1.30±0.78; t _(97)_ =2.41, p=0.018). Lower ratings were seen on sexual (Yes=1.00±0.0; No =1.14±0.46; t_(89)_ =2.96, p=0.004) and substance disorders (Yes=1.00±0.0; No=1.26±0.66; t_(89)_=3.66,p<0.001).A further analysis showed that there is no significant association between age and Axis I disorders.

### Axis II Disorders

The most reported personality traits were obsessive-compulsive personality, narcissistic personality and avoidant personality and the least reported conditions were schizotypal, antisocial and schizoid personality traits. The number of Axis II disorders (a score of 3 or greater) ranged between 1 and 9 per student, with an average of 1.89 traits (SD=2.10).

Further analyses showed that there are no significant associations between genders, age and psychotherapy and self-reported ratings of Axis II disorders. What is more, fifth year students reported higher levels of narcissistic traits compared to first year students (First year students= 1.29±0.69; Fifth year students=2.10±0.98; t_(76)_=4.19, p<0.001).

### Communality between Axis I and II ratings

The number of Axis-I disorders was significantly associated with Axis-II traits (r=0.59, p<0.001). Communal rating of axes indicated that most students (55.5%) were positive on both axes (4th category), while only 14.5% reported both axes were irrelevant (1st category). A significant association between academic year and communal rating of axes was observed (χ2_(3)_=13.2, p=0.004). This could be due to the fact that the majority of fifth year students (73.5%) were positive on both axes, compared to 38.2% for first year students. There was no statistically significant difference between gender and communal ratings (χ2_(3)_=4.4, p=0.22).

## Discussion

Results indicated that 55.5% of responding medical students admitted to the presence of some form of clinical symptomatology albeit with minimal severity, with both Axis I and Axis II disorders reported by over half of medical students surveyed. While these statistics may seem high, mean severity scores are relatively low, suggesting less of a presence of “full-blown” illness and more of a continuum of subthreshold conditions. These observations are significantly less than that reported by psychiatrists in a similar survey. In that survey, over 85% self-reported some form of clinical symptomatology albeit also with minimal severity.^21^ While these figures are significant, they reflect statistics similar to studies investigating incidence of subsyndromal phenomena in other populations.^15^^,^^16^While there was no difference in the presence or absence of Axis-II traits between males and females, females did self-report a higher number of Axis I disorders. More specifically, females self-reported higher levels of mood disorders, generalized anxiety disorder and social phobia. The phenomenon of higher levels of Axis I disorders in females was also noted in the previous cohort of psychiatrist participants. This observation reflects results from other larger epidemiological studies of mental illness in the community with regard to mood disorder where the incidence is considered to be close to double in females. It should also be noted that the distribution of affective disorders in females among study participants appears to mirror that of the general population in this age group.Although the questionnaire used in this study was based on subjective report thus introducing bias of self-report, it has value since to our knowledge this is the first time medical students have been directly asked regarding the presence of the full range of Axis I and II DSM-IV clinical phenomena. Thus the DSMIV criteria become the “standardised cut-off” for determining self-reporting. While it is fully recognized that to date this newly developed self-report instrument has no demonstrated reliability, validity or other biometric properties, considering the sensitive and preliminary pilot nature of the study, the observations were considered important enough to be reported in order to stimulate further research and awareness of the phenomenon in order to validate and confirm the findings. If true, the findings suggest that it would be important to further efforts in developing support programs for medical students during their studies coordinated by appropriate mental health professionals. Since this study investigated only medical students, it would be important in additional studies to investigate the phenomenon in other college student populations.Interestingly, students in their fifth year reported more Axis I disorders than those students in their first year (Table 1) and while there was no difference on overall reporting of Axis 2 traits, fifth year students did report that narcissistic traits were more common (Table 2). This latter finding requires further investigation since, while speculative, it may be suggested that the observation is associated with the phenomenon of "ethical decay". This relates to the phenomenon whereby medical students beginning medical school studies exhibit less cynicism and less tolerance of unethical behavior as compared to those later in medical training.^17^^,^^18^ It would be interesting in further studies to explore whether narcissism in this context would be associated with this “ethical decay” finding. Since previous investigation in medical students has shown lessening of disease distress and hypochondriacal concerns of medical disorders over the years of medical school as perspective of medical disease increases,^19^ in contrast it appears that subjective experience of psychiatric disorders may increase. In addition, several researchers have described that the percentage of medical students who report depressed mood increases over the years of medical school.^9^ While speculative, this may be related to increased stress and burnout resulting from studies and responsibilities during medical school training ^20^ as well as increased awareness of the existence of psychiatric conditions during studies of the subspecialty. Burnout would be particularly problematic since it is associated with self-reported reduced levels of patient care.^21^ One study indicated that 70% of medical interns meet criteria for psychiatric disturbance on at least one occasion during their internship year.^22^ A further consideration is the phenomenon of “medical student syndrome” (the tendency for students to see in themselves disorders they are learning about) which may also have influenced our findings. This latter factor is particularly an issue to consider since the students in their fifth year completed the questionnaire immediately after completing their clinical psychiatry rotation.Based on both the burden of studies and risk of burnout, medical students particularly in their later years of medical school and into their internship may be particularly vulnerable for psychiatric morbidity. Thus systems should be in place which recognize and remediate those in whom signs of psychiatric distress are discernible.^22^ Other considerations with regard to changes in approaches to medical education as well as evaluation procedures may be also related.^23^ Interestingly, while encounters with personal illness (mental or physical) may lead to lower scores on examinations and higher general anxiety, it has been reported that reflection on such experiences may improve professional attitudes towards patient care such as empathy and compassion.^24^

### The limitations of the study

While this was a preliminary pilot study, limitations include the relatively low sample number and the fact that students from only one medical school were surveyed. Furthermore, since this study was cross-sectional in nature seeking to compare students in years 1 and 5, it possibly introduces a cohort effect bias. Further studies therefore should be encouraged to examine the phenomenon in a prospective manner. Unfortunately the precise number and characterization of non-responders is unknown. While speculative it may be suggested that the medical students who are of most potential concern are those whose attendance and participation is poor. These students were not recruited for study participation since enrolment in the study was dependent on student attendance at lectures/tutorials. Further studies should attempt to reach out to this subpopulation of students using an approach creative in nature. While construct validity with the questionnaire is unproven, the same questionnaire has been used in two previously published studies and the questionnaire does appear to succeed in measuring what the researchers intended to measure i.e. the subjective perception of psychiatric conditions as defined in the accompanying key to the questionnaire. While this obviously does not qualify for diagnosis as would be the case if the participants had been examined, for example, by board certified psychiatrists, study observations do still provide some interesting information for further research of the subject.

## Conclusions

In summary, medical students report significant subjective levels of psychiatric morbidity albeit at low severity. Observations indicate that the phenomenon warrants attention, including programs specifically aimed at minimizing factors which may augment burnout levels in medical graduates.^22^ It has been stated that young doctors should be given the same care and support that we expect them to provide to their patients.^22^ The same should be extended to medical students in order to promote resilience and personal fulfilment, and for enhancement of professionalism and patient care.^20^ This would not only serve the needs of the student but also is in the long term interest of patients.

### Conflict of Interest

The authors declare that they have no conflict of interest.
